# Comparing the epidemiology of community- and hospital-associated *Clostridium difficile* infections in Northern Ireland, 2012–2016: a population data linkage and case–case study

**DOI:** 10.1017/S0950268819000414

**Published:** 2019-03-13

**Authors:** A. Maisa, G. Ross, N.Q. Verlander, D. Fairley, D.T. Bradley, L. Patterson

**Affiliations:** 1 Public Health Agency, Health Protection Service Northern Ireland, Belfast, Northern Ireland; 2 European Programme for Intervention Epidemiology Training (EPIET), European Centre for Disease Prevention and Control (ECDC), Stockholm, Sweden; 3 Statistics Unit, Statistics, Modelling and Economics Department, Public Health England, Colindale, England; 4 Department of Microbiology, Belfast Health and Social Care Trust, Belfast, Northern Ireland; 5 Centre for Public Health, Queen's University Belfast, Belfast, Northern Ireland

**Keywords:** Clostridium difficile, community-associated infections, hospital-associated, risk factors, ribotype

## Abstract

The burden of community-associated *Clostridium difficile* infection (CA-CDI) has increased. We aimed to describe the epidemiology of CA-CDI to inform future interventions. We used population-based linked surveillance data from 2012 to 2016 to describe socio-demographic factors, ribotype and mortality for all CA (*n* = 1303) and hospital-associated (HA, *n* = 1356) CDI. For 483 community-onset (CO) CA-CDI and 287 COHA-CDI cases, a questionnaire on risk factors was completed and we conducted a case–case study using logistic regression models for univariate and multivariable analysis. CA-CDI cases had lower odds of being male (adjusted odds ratio (AOR) 0.71, 95% confidence interval (CI) 0.58–0.87; *P* < 0.001), and higher odds of living in rural rather than urban settlement (AOR 1.5, 95% CI 1.1–2.1; *P* = 0.05) compared with HA-CDI cases. The distribution of ribotypes was similar in both groups with RT078 being most prevalent. CDI-specific death was lower in CA-CDI than HA-CDI (7% *vs.* 11%, *P* < 0.001). COCA-CDI had lower odds of having had an outpatient appointment in the previous 4 weeks compared with COHA-CDI (AOR 0.61; 95% CI 0.41–0.9, *P* = 0.01) and lower odds of being in a care home or hospice when compared with their own home, than COHA-CDI (AOR 0.66; 95% CI 0.45–0.98 and AOR 0.35; 95% CI 0.13–0.92, *P* = 0.02). Exposure to gastric acid suppressants (50% in COCA-CDI and 55% in COHA-CDI) and antimicrobial therapy (18% in COCA-CDI and 20% in COHA-CDI) prior to CDI was similar. Our analysis of community-onset cases suggests that other risk factors for COHA-CDI may be equally important for COCA-CDI. Opportunities to safely reduce antibiotic and gastric acid suppressants use should be investigated in all healthcare settings.

## Introduction

*Clostridium difficile* infection (CDI) is recognised as a hospital-associated (HA) infection responsible for significant morbidity and mortality [1–4]. Over the last two decades, there has been a significant reduction in the incidence of HA-CDI across the UK [5]. The reasons for this are multi-faceted but undoubtedly changes in prescribing and infection control behaviours have played a key role.

Unlike HA-CDI, the definition of community-associated (CA) CDI is more complex and very few countries have a national surveillance programme to promote improvement based on local intelligence. However, an increasing burden of infections that are CA has been observed from the available data [6–8]. This in part reflects a lack of understanding about risk factors which for CA-CDI, unlike HA-CDI [7], are still not well categorised [6, 9, 10]. This is influenced by both a relative lack of data and conflicting information about potential risk factors, such as antibiotic use [11].

While the incidence of CA-CDI has been increasing, there is limited information about patient outcomes. Studies on HA-CDI have identified CDI-specific mortality in the range of 7–42% [12]. However, similar information is lacking for CA-CDI, particularly in the UK setting. The virulence of the CDI infection is linked to the CDI ribotypes [7, 9, 13–15]. In most of the UK, ribotyping is conducted on a subset of cases which includes a random sample plus cases identified when an increased incidence is observed. This can skew the results to the most virulent strain and also means that a complete picture for community CDI is lacking [16].

In this study, we attempt to address these issues using a population dataset of infection and ribotype data to compare CA- and HA-CDI to: (1) describe the epidemiology of these infections, (2) describe CA-CDI case fatality and ribotypes and (3) compare risk factors for those with disease onset in the community with a view to informing preventive measures for CA cases.

## Methods

### Data sources

This was a population-based data-linkage study of all individuals with laboratory-confirmed CDI in Northern Ireland (NI) from 1 January 2012 to 31 December 2016 (*n* = 2807).

In 2012, the Public Health Agency (PHA) introduced an enhanced surveillance system for community-onset (CO) CDI. A questionnaire is completed for each CO-CDI case to collect information on potential risk factors including demographic data (age and sex), healthcare contact, selected medication use, travel history and infant contact. The CO-CDI dataset is matched to CDI infections reported to the PHA from Trust laboratories (*n* = 5) to produce a full population combined dataset of community and hospital onset cases. There are currently no private microbiology laboratories in NI. Ribotype information is available for the entire dataset.

For this study, the combined dataset was linked deterministically, using a unique identifier (the patient's health and care number) to the health card registration system which contains information on vital events such as deaths. We assigned the following to each CDI case based on the individual's postcode of residence: settlement of individuals which was classified into urban, intermediate and rural, using the Northern Ireland Statistics and Research Agency (NISRA) classification of settlements in NI, and the NI multiple deprivation measure 2010 (a composite measure of deprivation based on 52 indicators), which was used to assign a deprivation quintile ranging from most (1) to least deprived (5). We also identified case fatality (30-day all cause and CDI-specific).

### Case definition

**Fig. 1.**
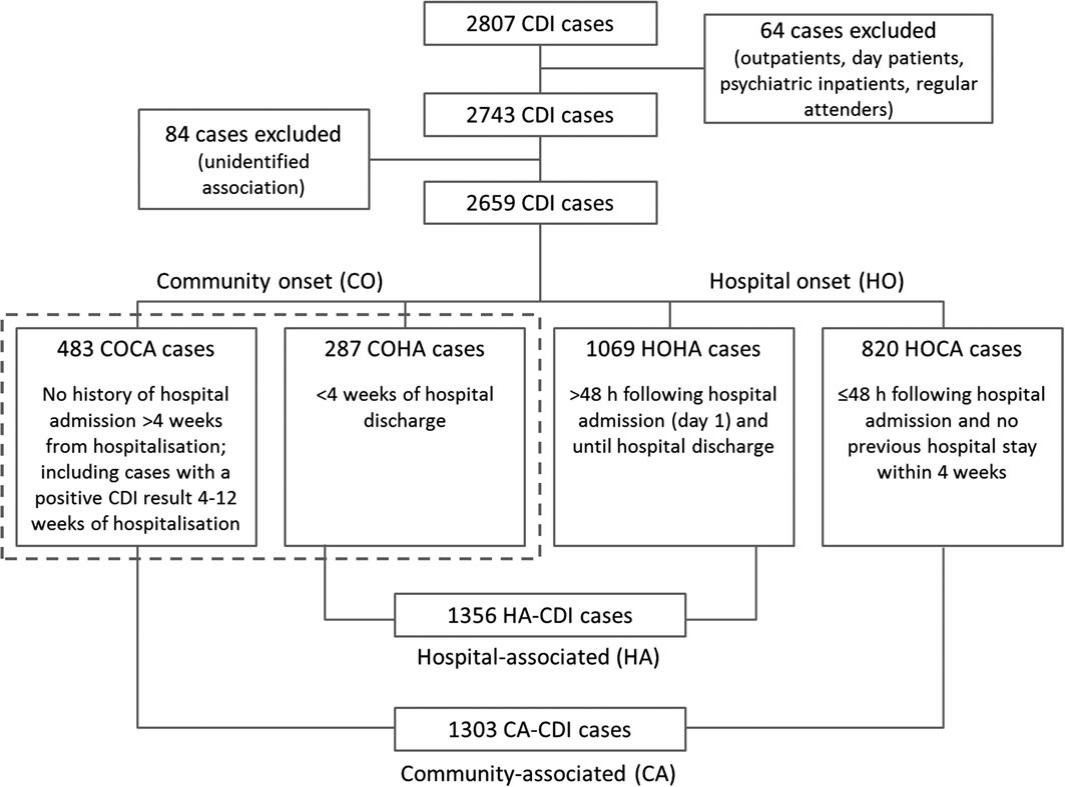
CDI surveillance case categories based on location and onset of symptoms highlighting the CDI population in Northern Ireland, 2012–2016. Dashed line comprises community-onset cases used in the case–case study. CDI, *Clostridium difficile* infection; CO, community onset; HO, hospital onset; CA, community-associated; HA, hospital-associated.

All CDI-onset categories were assigned according to the patient's location at the time the specimen was taken, which was used as a proxy indicator for date of symptom onset. The case was further categorised into either HA or CA to determine where the infection was likely acquired (Fig. 1).

For this analysis, outpatients, assessment and emergency patients, day patients, psychiatric inpatient and regular attenders were excluded (*n* = 64, Fig. 1) because of a lack of information regarding each individual's healthcare contact to accurately classify their association.

### Microbiology

Microbiological diagnosis of CDI was carried out according to current UK Department of Health guidelines [17] in medical laboratories accredited by the UK Accreditation Service. In brief, a two-stage algorithm used screening by either glutamate dehydrogenase enzyme immunoassay or molecular (PCR) testing to detect CDI, followed by toxin detection using enzyme immunoassay in screen-positive specimens. *C. difficile* strains were isolated from positive clinical specimens according to UK Standards for Microbiology Investigations methods [18]. Toxin gene detection and ribotyping of CDI isolates was done as described previously [19]. Double infections were defined as a CDI with separate ribotypes and assumed both ribotypes were present in similar amounts.

### Analytical strategy

For the first part of the analysis, we analysed trends, socio-demographic factors, case fatality and ribotype for all individuals aged 2 years and over with CA- (*n* = 1303) and HA-CDI (*n* = 1356).

CA-CDI incidence rates were calculated using mid-year population estimates (NISRA) and expressed as episodes per 100 000 population per year. HA-CDI were expressed per 10 000 occupied bed-days per year (data provided by the Hospital Information Branch, Department of Health, NI).

Of all cases, 29% were a recurrent (new episode of infection more than 28 days after the first positive specimen with negative specimen between both episodes) or continuing infection (episode of infection with no negative sample within 28 days) in the same patient, which were indistinguishable in our dataset. This clustering denied the assumption of independence between records and thus we examined the association between exposures and CA-CDI using a multilevel mixed-effects logistic regression model.

For the outcome data, we described the distribution of ribotypes (data available for 2123/2659 (80%) individuals, 375 (14%) could not be specified and 161 (6%) could not be isolated) for CA-CDI and HA-CDI. We also described all-cause and CDI-specific case fatality within 30 days (data available for 2654/2659 individuals; 99.8%). For both outcomes, we tested the association with CA-CDI compared with HA-CDI using logistic regression. We also compared the odds of CDI-specific death in CA-CDI using the most common ribotype as the baseline. This model was adjusted for a linear trend in age on the logit scale.

Secondly, we conducted a retrospective case–case study comparing risk factors for COCA-CDI *vs.* COHA-CDI. As enhanced risk factor information was only available for CO cases, this analysis was restricted to 483 COCA- and 287 COHA-CDI cases. We compared socio-demographic factors for COCA- and COHA-CDI and then examined the association between exposures and COCA-CDI using logistic regression. The assumption of linearity of the association between age and illness, on the logit scale, was assessed by fitting more complex polynomial functions and simplifying if these functions did not significantly improve the fit of the model. To build the multivariable model, variables with *P* < 0.2 in the univariate analyses were added in a forward stepwise fashion using a likelihood-ratio test at each step until all factors were significant at a 0.05 level or were substantial confounders, i.e. changed the odds ratio of one or more parameters remaining in the model by more than 20%. Age and sex were included as *a priori* confounders. The assumption of linearity for age was tested again in the final multivariable model. We excluded variables where collinearity was evident which included ‘having visited a hospital in the 4 weeks prior to *Clostridium difficile* diagnosis’ which was collinear with ‘having attended an outpatient appointment in the 4 weeks prior to *Clostridium difficile* diagnosis’.

We again tested the assumption of independence between CO cases using a multilevel mixed-effects logistic regression model. The estimates from this model were similar in magnitude, direction and significance to the logistic regression model and thus the simpler model is presented. We calculated odds ratios, 95% confidence intervals and likelihood ratio test *P*-values for both analyses.

In 2016, cases with a positive CDI result within 4–12 weeks of hospitalisation were classified as unknown origin (UNK) [20] compared with COCA classification prior to 2016. To assess the impact of this change, sensitivity analysis was conducted using data from 2012 to 2016 but excluding the unknown cases in 2016 (5.2%, *n* = 25) and comparing it to the primary analysis. Again, the estimates from both models were similar in magnitude, direction and significance and so the 25 cases were retained in the full analysis.

All analyses were conducted using Stata 2012 (StataCorp. 2011. Stata Statistical Software: Release 12. College Station, TX, USA: StataCorp LP.).

### Ethical consideration

The CDI data were reported by Health and Social Care (HSC) Trusts to PHA in its role as the body with statutory responsibility for health protection surveillance. In this case, research ethics approval was not required as a Data Access Agreement or equivalent contract states the terms of data use. The analyses were conducted using anonymised data. In addition, a specific Data Access Agreement was created between PHA and the HSC Business Services Organisation for assigned settlement, deprivation and the identification of deaths of people who had CDI.

## Results

### Incidence trends

**Fig. 2.**
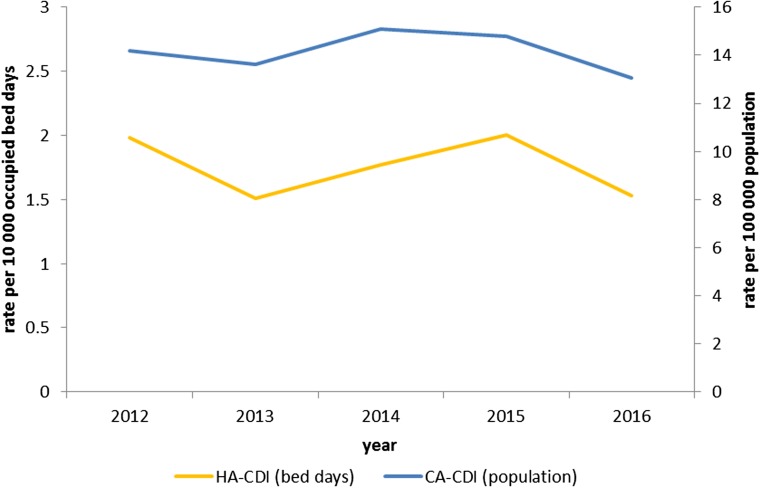
Rate of CDI per 100 000 population for community-associated (*n* = 1356) cases; and rate of CDI per 10 000 occupied bed-days for hospital-associated (*n* = 1303) cases in Northern Ireland, by year 2012–2016.

The overall CDI incidence was 28.9/100 000 population (2012–2016) with rates for CA-CDI of 14.2/100 000 population and for HA-CDI of 1.8 per 10 000 bed-days (2012–2016) (Fig. 2).

### Whole cohort analysis (HA-CDI *vs.* CA-CDI)

CA-CDI cases were significantly younger (adjusted odds ratio (AOR) 0.99; 95% confidence interval (CI) 0.99–1.0; *P* < 0.05) than HA-CDI and had lower odds to be male (65% were female; AOR 0.71; 95% CI 0.58–0.87; *P* < 0.001; Table 1).

**Table 1.** Descriptive epidemiology of all CDI cases by association with the community *vs*. hospital and univariable analysis, multilevel model analysis on a patient level

		CA-CDI (*n* = 1303)		HA-CDI (*n* = 1356)		OR (95% CI)	*P*-value^a^	AOR (95% CI)	*P*-value^a^
		*n*	%	*n*	%				
Age (years)	Range 25th, 75th Median	3–101 68, 86 79		4–103 70, 87 80		0.99 (0.99–1.0)	0.021	0.99 (0.99–1.0)	0.017
Sex (*n* = 2635)	Male Female	453 839	35 65	556 787	41 59	0.72 (0.58–0.88) ref	0.001	0.71 (0.58–0.87) ref	0.0007
Settlement band (*n* = 2619)	Rural Intermediate Urban	303 763 220	24 59 17	282 775 276	21 58 21	1.4 (1.1–1.9) 1.3 (0.97–1.6) ref	0.067	1.5 (1.1–2.1) 1.3 (0.96–1.6) ref	0.05
Deprivation (*n* = 2619)	Least 4 3 2 Most	231 241 231 300 283	18 19 18 23 22	279 249 261 256 288	21 19 20 19 22	0.85 (0.63–1.1) 0.99 (0.73–1.3) 0.91 (0.67–1.2) 1.3 (0.94–1.7) ref	0.11	0.81 (0.59–1.1) 0.9 (0.65–1.2) 0.8 (0.58–1.1) 1.1 (0.83–1.5) ref	0.14

a Likelihood ratio test *P*-value.

CA, community-associated; CDI, *Clostridium difficile* infection; HA, hospital-associated; OR, crude odds ratio; CI, confidence interval.

CA-CDI cases also had higher odds to live in a rural settlement band when compared with urban (AOR 1.5; 95% CI 1.1–2.1; *P* = 0.05).

### Ribotype analysis

**Fig. 3.**
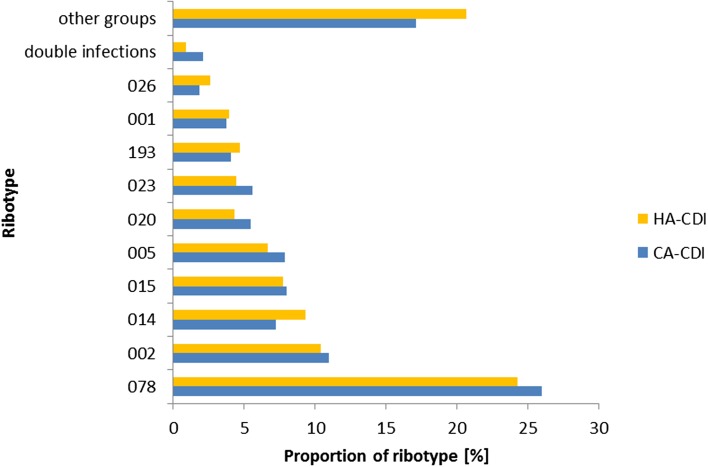
The relative frequency of CA-CDI ribotypes compared with HA-CDI ribotypes, in NI 2012–2016.

There was no difference in ribotypes between CA- and HA-CDI (Fig. 3) and the association between ribotype and the odds of CA-CDI was not significant (*P* = 0.12; data not shown). Indeed, the proportion of the top 10 ribotypes within CA- and HA-CDI was similar. The most prevalent ribotype for both groups was RT078 (26% *vs* 24%, *P* = 0.36). One exception was the prevalence of double infections which was higher for CA-CDI cases when compared with HA-CDI cases (1.04% *vs* 0.47%, *P* < 0.05). Of note, only six individuals had RT027 during the study period.

### Case fatality

CA-CDI cases had 51% lower odds for case fatality due to all-causes (OR 0.49; 95% CI 0.38–0.63; *P* < 0.0001) and 44% lower odds for a CDI-specific death (OR 0.56; 95% CI 0.4–0.8; *P* < 0.001) compared with HA-CDI.

Among CA-CDI, ribotype was not overall significantly associated with CDI-specific death within 30 days of specimen date (*P* = 0.2, adjusted for age). Using ribotype RT078 as the baseline, RT193 was seven times more likely to be associated with CDI-specific 30-day case fatality (AOR 7; 95% CI 1.3–37; *P* = 0.021; Table 2).

**Table 2.** Thirty-day CDI-specific mortality for CA-CDI by ribotypes and adjusted for age

CA-CDI (*n* = 1037)		30-day CDI-specific mortality (*n* = 64)		AOR^a^	95% CI	*P*-value^b^
		*n*	%			
Ribotypes	078	19	30	ref		0.2
	002	9	14	1.3	0.34–4.8	
	014	5	7.8	1.1	0.21–5.3	
	015	5	7.8	0.79	0.16–3.9	
	005	3	4.7	0.41	0.06–2.7	
	020	1	1.6	0.15	0.01–3.1	
	023	3	4.7	0.63	0.1–4.5	
	193	8	13	7.0	1.3–37	
	001	3	4.7	1.3	0.17–10	
	026	1	1.6	0.64	0.03–15	
Other groups		6	9.4	0.34	0.08–1.4	
Double infections		1	1.6	0.56	0.02–14	

a Adjusted for linear function of age.

b Likelihood ratio test *P*-value.

### Case–case study analysis (COCA-CDI *vs.* COHA-CDI)

In the univariate analysis, there was no difference in the age, sex, settlement band or deprivation status of COCA-CDI and COHA-CDI cases (Table 3).

**Table 3.** Risk factor analysis of CO-CDI cases by association with the community *vs*. hospital, univariate analysis and multivariable analysis (*n* = 629)

		COCA-CDI (*n* = 483)		COHA-CDI (*n* = 287)					
		*n*	%	*n*	%	OR (95% CI)	*P*-value^a^	AOR (95% CI)	*P*-value^a^
Age (years) (*n* = 770)	Range 25th,75th Median	4–101 73, 88 81		4–99 71, 87 81		1 (0.995–1.02)	0.3	1 (0.99–1.02)	0.16
Sex (*n* = 762)	Male Female	164 315	34 66	97 186	34 66	1 (0.73–1.4) ref	0.99	0.99 (0.69–1.4)	0.97
Settlement (*n* = 755)	Rural Intermediate Urban	122 277 75	26 58 16	67 167 47	24 59 17	1.14 (0.71–1.8) 1.04 (0.69–1.6) ref	0.83		
Deprivation (*n* = 755)	Least 4 3 2 Most	99 97 94 114 70	21 21 20 24 15	63 53 56 60 49	22 19 20 21 17	1.1 (0.68–1.8) 1.3 (0.78–2.1) 1.2 (0.72–1.9) 1.3 (0.82–2.2) ref	0.78		
Residence (*n* = 767)	Care home Hospice Own home	189 8 283	39 1.7 59	116 13 158	40 4.5 55	0.9 (0.67–1.23) 0.34 (0.14–0.85) ref	0.059	0.66 (0.45–0.98) 0.35 (0.13–0.92)	0.02
Antimicrobial therapy in previous 28 days (*n* = 646)		73	18	46	20	0.87 (0.57–1.3)	0.49		
Other resident on antibiotics (*n* = 602)		153	41	101	44	0.90 (0.65–1.3)	0.55		
Other person with diarrhoea at home (*n* = 703)		48	11	30	11	0.98 (0.60–1.6)	0.93		
Visited hospital in previous 4 weeks (*n* = 598)		44	11	72	37	0.21 (0.14–0.33)	<0.0001		
Outpatient appointment in previous 4 weeks (*n* = 640)		105	24	66	32	0.68 (0.47–0.99)	0.043	0.61 (0.41–0.9)	0.01
Travel outside NI (*n* = 704)		11	2.5	7	2.6	0.95 (0.36–2.5)	0.91		
Contact with infant <2 years (*n* = 605)		8	2.1	8	3.5	0.61 (0.23–1.6)	0.33		
Gastric acid suppression (*n* = 732)	PPI H2 PPI + H2	230 20 2	50 4.3 0.43	151 6 0	56 2.2 0	0.82 (0.6–1.1) 1.8 (0.7–5.6) 1.3^b^(0.1-Inf)	0.18^c^		

a Likelihood ratio test *P*-value unless specified otherwise.

b Median unbiased estimate from exact logistic regression.

c Fisher's exact *P*-value.

PPI, proton pump inhibitor; H2, histamine-2 receptor antagonist.

COCA-CDI had 39% lower odds of having had an outpatient appointment in the previous 4 weeks compared with COHA-CDI (AOR 0.61; 95% CI 0.41–0.9, *P* = 0.01). This patient group also had lower odds of being in a care home or hospice when compared with their own home, than COHA-CDI (AOR 0.66; 95% CI 0.45–0.98 and AOR 0.35; 95% CI 0.13–0.92, *P* = 0.02).

The proportions exposed to various risk factors were similar in both COCA- and COHA-CDI (Table 3), including 18% and 20% of both groups receiving antimicrobial therapy in the previous 28 days. Similarly, prescribing of gastric acid suppression therapy in the community was equally high in both groups (55% *vs*. 58%, for COCA and COHA, respectively).

## Discussion

Our analysis of a population cohort of CA-CDI has shown that these cases are likely to be younger, female and live in rural areas. The most prevalent ribotype in CA- and HA-CDI continues to be RT078. We also observed lower odds of CDI-specific death within 30 days in CA-CDI cases. We can speculate that the reasons for this may include the likely higher vulnerability, frailty and comorbidity in the inpatient hospital population. In terms of risk factors among CO cases, this analysis has highlighted more similarities than differences between COCA-CDI and COHA-CDI. In both groups, we observed a high prevalence of modifiable risk factors, such as PPI use and exposure to antibiotics, which is known to increase the risk of CDI [7].

Our first objective was to describe the epidemiology of CA-CDI using a population dataset. We showed that almost half of CDI cases in NI are CA, and while the HA-CDI rate was stable during the study period, it has decreased over the last decade [21]. Despite this, the rate of CA-CDI was within estimates summarised by one study [22] and lower when compared with another study that used an equivalent case definition [8]. However, considering that underdiagnosis of CDI in the community is likely [11], the burden of disease in the community might even be greater than estimated. The biggest challenge for making comparisons remains the difference in case definitions used as well as the difference with denominators, such as whether rates are presented per population or bed days [23].

Using all CDI cases, we identified that CA-CDI had higher odds of being from a rural settlement compared with urban and that the most prevalent ribotype is RT078, which is commonly isolated from cattle and pigs [24, 25]. Farming is one of the main industries in NI and despite the lack of occupational information of the patients, we hypothesise that it may be exposures like farm or animal contact that have a role in CA-CDI here. This has been discussed in other studies [14, 26, 27].

Our second objective was to compare both ribotype and CDI-specific case fatality for CA-CDI. Here, with the benefit of ribotype data for almost all CDI cases, we showed an equal diversity of ribotypes among CA- and HA-CDI, which has been described previously [9, 13]. We found that RT078 [19, 26] was the most prevalent type, which has also been observed in Scotland (19% RT078 CA-CDI, 13% all CDI) [28]. An interesting finding was the higher proportion of double infection in CA-CDI. Double infections may play a role in horizontal gene transfer by exchanging virulence factors between multiple ribotypes [29]. This may pose a risk for the emergence of more virulent ribotypes. Despite low numbers, the higher proportion of double infection in CA-CDI warrants further investigation.

We showed that people with CA-CDI had lower odds of dying of CDI-specific causes within 30 days of their specimen compared with people with HA-CDI, which is consistent with a previous study using a similar case definition [8]. We hypothesise that the lower case fatality rates in CA-CDI may reflect these individuals having less co-morbidity and being slightly younger compared with HA-CDI. Indeed, RT193, which belongs to the same clade 5 of ribotypes as RT078, was shown to pose an even higher risk for CDI-specific death among CA-CDI when compared with RT078 in our study. Increased 14-day mortality for CDI in a hospital context has been observed for clade 5 ribotypes previously [30].

The last objective of this study was to try and identify risk factors for COCA-CDI when compared with COHA-CDI. We started with known risk factors for HA-CDI with a view to trying to discount those that were not relevant in the community setting. Our study showed that there was no difference in the odds of COCA-CDI when we look at antibiotic prescribing. That is, in both COCA-CDI and COHA-CDI, approximately 20% of patients with CDI had been exposed to antibiotics (in the preceding 28 days). This contrasts with another study, which showed that antimicrobial therapy was less frequent in CA-CDI compared with HA-CDI, but similar to our findings at 28% [31]. There is strong evidence that antibiotic stewardship programmes can be used to reduce the risk of CDI [32]. Given the lack of difference between COCA-CDI and COHA-CDI, we suggest that antibiotic stewardship in the community could be a focus for improvement efforts. However, the development of targeted initiatives will require further investigation into the type and duration of antibiotic treatment, which was not available for this analysis.

Another modifiable factor which showed no difference between COCA-CDI and COHA-CDI was the use of PPIs. Indeed, half of patients received PPIs (50% and 56%) in the community and hospital setting. This is concerning given the potential role of PPI use in promoting CDI [33]. This has been supported by a recent meta-analysis of PPI use and the risk of developing CDI. The authors concluded that there was a need to establish guidelines for the use of PPIs which may help with the control of CDI [34]. It is clear that these guidelines would be appropriate in both the community and hospital setting.

We found that 28% of community-onset cases of CDI were not known to have received either antibiotics or PPIs, which has been observed by others [22]. Possible sources could be transmission via food, animals or other people [35], and it is possible that some exposures were not known to those providing risk factor information. There is increasing evidence linking CDI to environmental sources including water and food [25, 36–38], but also pets [39], farming [40] and asymptomatic carriers [35]. While these factors were not captured in the enhanced surveillance programme, they should be considered in the future.

This study has a number of strengths. We used a full population cohort of CDI patients with an unbiased sample of ribotype data and linked CDI-specific case fatality information. However, there are a number of limitations which influence the interpretation of the data. We have categorised CDI cases according to whether or not they had a hospital admission which will underestimate true healthcare contact defined by contact with primary care, secondary care (those that were not admitted) and care homes. We felt this was the best approach to classifying cases given the complexity of the patient journeys and the limited information collected. Despite this limitation, our results were comparable with previous studies [8, 22].

The classification of the indeterminate group of unknown origin, as per latest definition by the European Centre for Disease Prevention and Control [20], was not available for the whole dataset as the case definition was only implemented in NI in 2016. We did assess the impact of this and concluded that it was not substantial. However, as the numbers of these cases were small in 2016, future studies are needed to assess if CDI cases with unknown origin are more likely to be similar to CA- or HA-CDI.

The risk factor data were only available for CO cases which were not representative of the whole cohort. This means that the findings from the case–case study cannot be extrapolated to the whole population but must be limited to CO cases only. This also meant that for the whole cohort analysis, we were unable to adjust for other potential confounders, such as co-morbidities. Our analysis of ribotype-specific case fatality was adjusted for age, but there could be residual confounding caused, for example, by co-morbidities, so the data should be interpreted in this context. We described a higher proportion of infections with two ribotypes in the community. However, these may be underestimated as laboratory detection methods are more likely to detect the more prominent ribotype in a double infection. Therefore, our results on infections with two strains may under- or overestimate the effect of a double infection on CA-CDI. This should be explored in future studies. Nevertheless, it is difficult to assess the level of care patients in different residence settings are receiving. Also, the duration and type of antibiotic treatment were not available in our study. Similarly, we identified a number of recurrent infections in our analysis. We accounted for these using multilevel multivariable regression, which minimised the possibility of introducing a selection bias by choosing one episode for each patient. However, it is possible that individuals with recurrent infections may differ to those with a single infection in terms of risk factors and outcomes. We therefore recommend a further analysis to examine risk factors and outcomes for individuals with recurrent CDI. Finally, because of the availability of surveillance data, we were unable to classify outpatients, assessment and emergency patients, day patients, psychiatric inpatient and regular attenders using our case definitions. The exclusion of these cases may potentially introduce a selection bias; however, we feel that because they represent a small proportion of all cases (5%), this effect is likely to be minimal.

## Conclusions

CA-CDI accounts for half of all CDI in NI. While CDI-specific case fatality is lower in this group than in those whose infections are likely HA, action is required to reduce the burden of preventable CA-CDI. The lack of difference in the exposure to known risk factors for CDI, particularly for modifiable exposures such as antibiotic and PPI use, suggest these are equally important for CA-CDI. In response to the high level of prescribing among COCA-CDI cases, we recommend a further data linkage study on different classes of antibiotics for possible targeted interventions. Opportunities to safely reduce antibiotic and gastric acid suppressants use should be investigated in all healthcare settings.
